# *Pichia pastoris* (*Komagataella phaffii*) as a Cost-Effective Tool for Vaccine Production for Low- and Middle-Income Countries (LMICs)

**DOI:** 10.3390/bioengineering8090119

**Published:** 2021-08-31

**Authors:** Salomé de Sá Magalhães, Eli Keshavarz-Moore

**Affiliations:** Department of Biochemical Engineering, University College London, Gower Street, London WC1E 6BT, UK; s.magalhaes@ucl.ac.uk

**Keywords:** vaccines, LMICs, expression platforms, *P. pastoris* (*Komagataella phaffii*)

## Abstract

Vaccination is of paramount importance to global health. With the advent of the more recent pandemics, the urgency to expand the range has become even more evident. However, the potential limited availability and affordability of vaccines to resource low- and middle-income countries has created a need for solutions that will ensure cost-effective vaccine production methods for these countries. *Pichia pastoris* (*P. pastoris*) (also known as *Komagataella phaffii)* is one of the most promising candidates for expression of heterologous proteins in vaccines development. It combines the speed and ease of highly efficient prokaryotic platforms with some key capabilities of mammalian systems, potentially reducing manufacturing costs. This review will examine the latest developments in *P. pastoris* from cell engineering and design to industrial production systems with focus on vaccine development and with reference to specific key case studies.

## 1. Introduction

Infectious diseases have a significant global economic and societal impact. As a result, during the last two centuries, we have witnessed the remarkable success of vaccination reducing the burden of infectious diseases [1]. Although vaccination continues to be the most successful and effective mechanism against infectious pathogens, a plenitude of factors, such as global population growth, age distribution, traveling habits, as well as climate changes and antibiotic resistant bacteria, are causing the emergence of old and new pathogens with the risk of becoming pandemic threats [2,3]. Therefore, the global demand for vaccines is growing. Regardless of the approach that is taken, the development of an effective vaccination strategy that induces long-term immunity is a common aspect and pivotal for the success of a vaccine. However, making effective vaccines against our most challenging diseases such as AIDS, malaria, Ebola, and dengue, has proved to be very difficult to achieve, mainly due to the complexity of target pathogens. Recent efforts have been made to produce a vaccine against these, and efficacy rates of nearly 50% have been reported [4]. For example, there are several vaccine candidates in clinical trials against malaria RTS/S [5], HIV (RV144, [6]), and the Ebola virus (VSV-EBOV, [7]).

**Figure 1:**
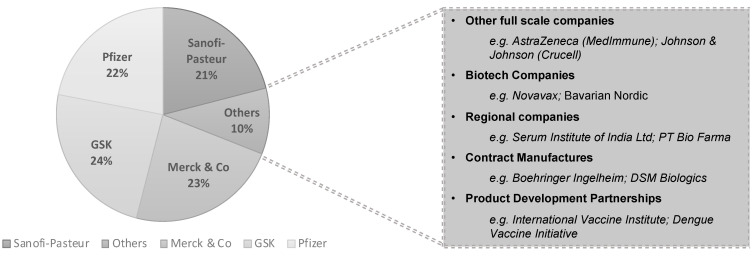
Global vaccine leaders market shares. Adapted by permission of Springer Nature Customer Service Centre GmbH: Nature [*Nat. Rev. Drug Discov.*] [8], (Shen, A.K. and Cooke, M.T. Infectious Disease Vaccines, 2019), [copyright] [10].

Despite huge impact on human health, the vaccine industry is relatively small if compared with the pharmaceutical industry. In 2017, the vaccine sector represented 3.5% ($28 billion) of the worldwide prescription drug sales, and it is expected to reach $81.27 billion by 2027, growing at a compound annual growth rate (CAGR) of 8.7% [8]. To date, 90% of the world market share is divided by only four companies: GlaxoSmithKline, Merck & Co, Sanofi-Pasteur, and Pfizer (Figure 1). The vaccine industry scenario is changing, not only with the growth of new markets in emerging economies, and the need for new vaccines for the developing world, but also given the imperative for speed to tackle the emergency situation we are living due to the COVID-19 pandemic [9].

Traditional vaccines are based on formulations comprising either live, attenuated or killed, or inactivated bacteria or viruses. Although its success, there are still many issues associated with these types of formulations. Namely, their ineffectiveness in the case of the live attenuated ones, the possibility to lead to severe disease in immunocompromised people, and the production costs and administration makes these vaccines difficult to use for mass immunization. Furthermore, with the increased demand of regulatory authorities, such as the United States Food and Drug Administration (FDA), the European Medicines Agency (EMA), and the World Health Organization (WHO), requiring precisely specified compositions and working with whole-cell vaccines becomes particularly challenging due to their undefined molecules. Over the last three decades, there has been a trend towards new vaccine formulations that comprise defined antigenic components, such as polysaccharides, nucleic acids, or proteins. An example that is of growing importance is that recombinant proteins are gaining, due not only to their wide application as biopharmaceutical products, but also to their proven safety record (non-infectious) and highly immunogenicity.

Such vaccine manufacturing processes rely on the use of recombinant proteins. Among many conventional and emerging cell-based systems for protein production, expression of recombinant protein-based biopharmaceuticals has been achieved using bacteria, mammalian cells, yeast, insect cells, transgenic plants, and transgenic animals, and, more recently, cell-free systems. The choice of the most appropriate expression system to gain scalability and high yield, as well as to reduce cost and time, should be previously identified, and normally requires optimization at the genetic and cultivation level which is product-dependent. A comparison of the expression systems as well as examples of recombinant protein-based biopharmaceuticals is presented in the Table 1.

In this review, we will focus on the use of yeasts as recombinant protein expression systems, more specifically on the application of *P. pastoris* as one of the most promising candidates for expression of heterologous proteins. This methylotrophic yeast is also considered a unique host for the expression of subunit vaccines which could significantly affect the growing market of medical biotechnology. A SWOT analysis to use *P. pichia* as platform technology is shown at Table 2.

## 2. Versatility of Yeasts as an Expression System Platform

The use of yeasts is widespread across all different areas of biotechnology, from white (industrial) to red (medical) biotechnology. Their unique characteristics, such as being generally regarded as safe (GRAS), having a relative fast growth, being simple to manipulate genetically (similar to prokaryotes), having scalable fermentation with short generation time, and eukaryotic type post-translational modifications (PTMs) [14,15], make them ideal for the production of biopharmaceuticals. Yeast have been used for the production of recombinant therapeutic proteins (e.g., insulin, human hemoglobin, and parathyroid hormone); immunomodulation and anticancer agents (e.g., cell wall glucan, 𝛽-glucan, and peptide-modified glucan particles (PcGPs)); amine-modified glucan particles (amGPs); and human glycoproteins (e.g., *N-*glycosylation modification: Man5GlcNac2, *O-*glycosylation modification, i.e., gps without high mannose, and *N-*glycosylation modification, i.e., GlcNAcMan5GlcNac2) [14,16]. In addition to the examples above, yeasts have also found a special place within vaccine production (Table 3). In particular for vaccine manufacturing, yeasts are versatile platforms, expressing different forms of antigens. This expression, more importantly, can be performed in high quantities and at low costs. *Saccharomyces cerevisiae (S. cerevisiae)* and *P. pastoris* are strong model systems for vaccine development for both humans and animals. This is due to the availability of a complete genome sequence; well-established genetic, inherent natural adjuvant; and their non-pathogenic nature. However, due to its shorter and less immunogenic glycosylation pattern together with higher cell density growth and higher secreted protein yield, *P. pastoris,* has emerged as the most popular alternative host in comparison with *S. cerevisiae*.

## 3. *P. pastoris* as a Powerful Protein Production Host System

*Pichia* as a host system has many advantages but also challenges as shown in Table 4.

From a manufacturing point of view, by using *P. pastoris*, a high cell density can be reached, allowing controllable processes. The cell lines are genetically highly stable (proteins are correctly folded in the endoplasmic reticulum) and present high resilience to contamination. It is well known that secreting low levels of endogenous proteins allows for the implementation of a simple recovery stage, saving time and cost associated with purification steps. Over 500 heterologous proteins have been successfully expressed using this host, with several being approved for human and commercialized use (e.g., Kalbitor^®^ (Burlington, MA, USA), from Dyax for the hereditary angioedema treatment, the Insugen^®^ (Bangalore, India), from Biocon for diabetes therapy, the Shanferon™ (Bengaluru, India), from Shantha/Sanofi for hepatitis C and cancer treatment, the Nanobody^®^ ALX00171 (Ghent, Belgium), from Ablynx for respiratory syncytial virus (RSV) infection treatment) [27,28].

Nonetheless, despite the impressive advantages of using this methylotrophic yeast, not every protein of interest is produced or secreted by *P. pastoris* in high titres that can meet commercialization demands. This is particularly relevant if the target-expressed proteins are hetero-oligomers, membrane-attached, or prone to proteolytic degradation [27]. To address this, over the years, we have been witnessing numerous activities related to the improvement of the expression of such complex proteins. The strategies to improve the efficiency of recombinant protein expression are variable, and mostly present an interplay between strain engineering (e.g., gene optimisation/synthesis; plasmid construction; host strain) and process engineering (e.g., different fermentation mode; cultivation parameters) (Table 5).

With the increase in product titre achieved during fermentation high cell density operation, the focus turns to the development of a rapid and efficient primary recovery and purification operations.

During this stage, efficient primary recovery of secreted products from the cell culture is crucial for minimizing manufacturing costs as product loss, which has a significant effect on the overall product yield. Recovery processes that consist of a low number of purification steps generally have lower capital and operating costs, and the overall yield is also expected to increase with a decreasing number of downstream processing steps.

Complementary methodologies, such as high-throughput processing, single-use systems modelling, continuous downstream processing, and the integration of upstream processing (USP) and downstream processing (DSP), have enhanced the efficiency of this expression system and accelerated process development. In Table 6, examples of different strategies to improve the efficacy of downstream processing are compiled. Hence, reaching ideal conditions is nonetheless straining and protein-specific, with the necessity of adjustment of the optimal conditions case-by-case [27,28]. Finding the quickest and cost-effective process is detrimental for the expression of heterologous proteins for clinical applications. Thus, the rationale behind such goals means considering the endpoint application before deciding on the strategy for the expression of heterologous proteins and, accordingly, selecting the expression system characteristics.

Legend: chromatography microplates (Chromafil^®^ Multi96), single-use centrifugation systems (Ksep^®^), single-use tangential flow filtration systems (ARTeSYN’s^®^), statistical software (Jmp, Design-Expert, Modde), integrated scalable cyto-technology (InSCyT).

### 3.1. Generalised Research Application Examples of the Overall Improved Features of P. pastoris

*P.pastoris-*based expression systems to offer a broad range of possibilities for the expression of secreted, endogenous, or membrane-bound proteins thanks to a combination of various plasmid backbones, selection markers, promoters, and fusion sequences that are introduced into dedicated host strains. Some examples of heterologous proteins, with biopharmaceutical importance produced in *P. pastoris*, and taking into consideration the features of different hosts, are lipase (X-33, WT/Mut^+^) [44], anti-CTL4 scFv Ab (GS115, His4/Mut^+^) [45], human insulin (SuperMan5) [46], recombinant hepatitis C (HCV), and glycoproteins E1E2 (SMD, His^+^/Mut^+^) [47]. Nonetheless, to achieve an optimal level of expression from host strain engineering, vector design, cultivation process, as well as post-translational modification, has been carried out as follows [48].

#### 3.1.1. Strain Engineering

Genetic engineering offers a powerful tool to increase productivity and improve product characteristics. Tools and methods for targeted genome modifications are of great interest. An example is the CRISPR–Cas9 method, which has been widely used for gene editing with the aim of gene mutation, insertion, and deletion in various species. This approach is beneficial to overcome limitations related to the reduced availability of selective markers and low efficiency of homologous recombination in *P. pastoris* [49,50,51]. As an example, Weninger and co-workers [52] described a CRISPR–Cas9 genome editing method where an effective gene mutation, deletion, and replacement was achieved by placing the human codon-optimized *cas* gene and the ribozyme-mediated guide RNA (gRNA) cassettes on the same plasmid containing the autonomously replicating sequence (ARS). In a different study, a CRISPR–Cas9-mediated multi-loci gene integration method was developed with efficient gRNA targets in *P. pastoris* [53]. Using this method, the authors showed that multiple gene cassettes can be simultaneously integrated into the genome without employing selective markers. This will benefit the pathway assembly of complicated pharmaceuticals and chemicals expressed in *P. pastoris*. With the aim of obtaining high editing efficiency and shortening the experimentation period, Yang et al. [54] have developed a CRISPR–Cas9 system with episomal sgRNA plasmid, showing that high multicopy gene editing and stable multigene editing were obtained without a sharp decline caused by multi-sgRNA.

One of the several advantages of using *P. pastoris* as an expression system is its ability to secrete a protein, which, for large-scale industrial production, offers the advantage of simple and efficient downstream purification, thus avoiding costly cell rupture, denaturation, and refolding. However, it is very challenging to optimize protein secretion due to the multiple steps involved during secretion and a lack of genetic tools to fine tune this process. Toolkits have been developed to standardized regulatory elements specific for *P. pastoris*, which allows for the tuning of gene expression as well as the choice of protein secretion tag [55]. After assessing the expression and secretion efficiency of 124 constructs that combined different regulatory elements with two fluorescent reporter proteins (RFP, yEGFP), the authors demonstrated that this approach is valuable for generating diverse secretion libraries when searching for optimal expression conditions, and ultimately for the creation of efficient microbial cell factories.

The improvement of secretion signals is also critical for the secretion of certain proteins from *P. pastoris*. One of the most commonly employed secretion signals is the *N-*terminal portion of pre-pro-α-factor from *S. cerevisiae* α-MF. A study by Barrero et al. [39] addresses both limitations of the pre-pro-α-factor secretion signal. To poorly overcome translocation of proteins that fold in the cytosol, i.e., poorly secreted, as well as the possibility of proteins self-association, where the α-factor pro region can potentially cause aggregation hampering export from the endoplasmic reticulum (ER), the authors have engineered a hybrid secretion signal consisting of the *S. cerevisiae* Ost1 signal sequence, which promotes co-translational translocation into the ER followed by the α-factor pro region. Another approach being used to improve the α-MF is site-directed mutagenesis of the pre-pro region [56], where the effect of various deletions and substitutions on expression was performed to evaluate the stretches of amino acids that can be removed to improve secretion and, consequently, to generate a model to elucidate the structure–function relationship it has for secretion in *P. pastoris*. Improving protein secretion can be accomplished by optimizing the Kex2 P1′ site, enzyme that is responsible for removing the signal peptides from pre-proteins and releasing the mature form of secreted proteins. Recombinant proteins were greatly influenced by Kex2 P1′ site residues and the optimized P1′s amino acid residue could largely determine the final amount of secretory proteins synthesized and secreted. This could also be achieved by the introduction of additional *Kex2* copies into yeast genome [57].

*P. pastoris* harbors several strong or weaker promoters that can be exploited to drive heterologous expression of recombinant genes, both in an inducible or constitutive fashion. On the one hand, the most used is the promoter P*_AOX1_*. This inducible promoter is tightly repressed by glucose and strongly induced by methanol [58], allowing the cells to use methanol as the sole carbon source. The differences in methanol utilization by this promoter are dependent on the strain phenotype, e.g., Mut^+^ (methanol utilization plus), Mut^S^ (methanol utilization slow), and Mut (methanol utilization minus), and have an impact on the enhancement of recombinant protein expression [59]. On the other hand, for a constitutive expression performance, the P*_GAP_* promoter is used. This promoter does not require methanol for induction. This feature makes strain growth more straightforward since no switch of carbon source is required, eliminating the hazards and costs related to the storage and delivery of large volumes of methanol [60]. However, the challenge associated with this promoter is the toxic effect that the recombinant protein expression can have on the host strain. In both cases, promoter libraries have been designed, constituting a valuable addition to the genetic toolbox for fine-tuned gene expression in *P. pastoris* [60,61].

Besides the genetic modifications, other approaches have been sought to improve the best features of the promoters. Várnai et al. [62] evaluated the expression of endoglucanases in *P. pastoris* under control of the GAP promoter. The particularity of this study was the use of PichiaPink™ expression system, normally applied to allow rapid clone selection and deliberately used to generate multi-copy clones, employing the methanol inducible AOX1 promoter to ensure high protein expression levels, being switched by the GAP promoter, pPink-GAP. The purpose of this new construct was to simplify enzyme production compared to methanol inducible expression systems. Reasonable protein levels after optimizing cultivation conditions were achieved. In the work performed by Chang et al. [63], a platform was developed as an alternative approach to express target genes driven by P*_AOX1_* under glycerol starvation, thereby eliminating the potential risks of methanol without losing the advantages of P*_AOX1_* promoter. The authors have applied a strategy to reprogram regulatory networks in *P. pastoris*, by inserting a synthetic positive feedback circuit of Mxr1 driven by a weak *AOX2* promoter P*_AOX2_*. Mombeni et al. [64] has developed a new powerful promoter, pMOX, for recombinant protein production, where the promoter region of MOX, isolated from *Hansenula polymorpha,* was replaced with the pAOX1 in the pPINK-HC plasmid in protease-deficient and wild-type *P. pastoris* strains. In this study, the authors investigated the effect of protease deficiency trait on expression levels as well as the gene dosage. The obtained results suggested that pMOX is a satisfactory alternative for pAOX1. One of the main disadvantages of both promoters, P*_AOX1_* and P*_GAP_*, is their lack of tunability. Taking that into consideration, a novel bi-directional promoter system, allowing tunable recombinant protein production in *P. pastoris,* was developed by Rajamanickam et al. [65]. The system was created by combining a modified catalase promoter system (P_DC_; derepressible and inducible) and the traditional P_AOX1_. In response to the available C-sources, this bi-directional promoter system offers a high degree of freedom for bioprocess design and development, making bi-directional promoters in *P. pastoris* highly attractive for recombinant protein production.

Additionally, Vogl and Glieder have provided a review of biotechnology-relevant aspects of *Pichia pastoris* promoters, including P_AOX1_, and alternative promoters such as P_TEF1_, P_PHO89_, P_THI11_, or P_AOD_ [66]. The group have also created the first synthetic yeast core promoter for *Pichia*, based on natural yeast core promoters. Short core promoters can directly be added on a PCR primer, facilitating library generation to obtained variable expression yields [34,61,67].

#### 3.1.2. Process Engineering

Another feature to consider for heterologous protein expression is the host strain itself. Recently, Brady et al. [68] have shown that the transfer of two beneficial mutations identified in X-33 into Y-11430 resulted in an optimized base strain that provided up to four-fold higher transformation efficiency and three-fold higher protein titers while retaining robust growth. The authors have proved that comparative genome-scale analysis of *P. pastoris* variants enables the selection of an optimal base strain as well as the prediction of performance that can guide selection of an optimal host. The main differences between *Pichia* strains, relies on their auxotrophic behavior, deficiencies in endogenous proteases, and, more recently, their capacity in performing “human-like” *N-*glycosylations. Engineering *P. pastoris*, i.e., making this host capable to produce human-like glycoproteins with high homogeneity, increases the importance of this yeast as a workhorse for recombinant protein production. Moreover, the *N-*glycosylation pattern is extremely relevant in the field of therapeutic proteins, due to molecular stability, solubility, in vivo activity, serum half-life, and immunogenicity. Therefore, over the years, several attempts have been made to humanize the yeast cell through glycoengineering [27,69,70,71].

Recently, Wang and co-workers [72] published a study about the homogeneous production and characterization of recombinant *N*-GlcNAc-protein. Their work consisted on the construction of a *P. pastoris* system expressing truncated *N*-GlcNAc-modified recombinant proteins through introducing an ENGase isoform (Endo-T), which possesses powerful hydrolytic activities towards high-mannose-type *N*-glycan in an intracellular environment, into different subcellular fractions. The achieved results demonstrated that the location of Endo-T in different subcellular fractions affected their hydrolytic efficiencies, corroborating the findings previously achieved by Claes et al. [73]. Therefore, the developed system provides a prospective platform for mass production of increasing novel glycoprotein drugs. Liu et al. [74] developed a methodology for the expression of antibodies from yeast to enable the large-scale synthesis of antibodies for further manipulation of the glycan moiety. Moreover, they have demonstrated the use of endoglycosidases for remodeling glycans on Herceptin, a monoclonal antibody used in the treatment of breast cancer to optimize its effector functions, especially the antibody-dependent cellular cytotoxicity (ADCC).

As mentioned previously, besides the strain engineering, process engineering is also a critical step to enhance the efficiency of recombinant protein expression. Process development strategies, along with genome-wide sequencing, leads to a rapid improvement of fermentation processes. Additionally, reaching the optimal expression conditions is strain- and target-protein-dependent, in particularly, for the secreted proteins, as the media composition may affect their genetic stability. Therefore, high throughput screening (HTS) of expression conditions is often required to identify the most optimal conditions. As an example, Kaushik et al. [75] described the possibility of using a microscale cultivation strategy for rapid high-throughput screening of *P. pastoris* clones, media optimization, and high-throughput recombinant protein production; a strategy that can be applied to other suspension cultures, with some modifications.

A different approach is the design of experiments (DoE) and artificial intelligence-based technique to maximize the production of huIFNα2b in recombinant glycoengineered *P. pastoris* [76]. The culture medium optimization, using this technique, resulted in enhanced production of huIFNα2b in glycoengineered *P. pastoris* at both the shake flask and bioreactor level. Moreover, the purified huIFNα2b was found to be N-glycosylated and biologically active, which is of high interest for the application in cancer therapy. Matthews and co-workers [77] designed a rich defined medium (RDM) for *P. pastoris* by systematically evaluating nutrients of increasing complexity and identifying those that are most critical for its growth. The evaluation was complemented by employing transcriptomics to gain deeper insights into the underlying metabolic processes. The authors have shown that the developed medium lead to yields titres comparable to, or higher than, those in standard complex medium, and that the usefulness of transcriptomics to accelerate process development for new molecules.

Continued progress in this area could lead to a new model for low-cost production of high-quality biologic drugs [78]. Proteolysis of a subunit rotavirus vaccine candidate using fed-batch fermentation, was evaluated. In this study, the authors developed a novel acidic pH pulse strategy to minimize proteolysis, thus improving the fermentation process conditions, and, in combination with an early harvest time, were able to obtain most of the material as full-length. Product quality was improved and maintained throughout the purification process. A huge amount of biopharmaceutical drugs and industrial enzymes have been successfully produced by fed-batch high-cell-density fermentation (HCDF) using *P. pastoris.* However, selecting a suitable HCDF strategy is not a straightforward process. One major bottleneck is the manipulation of the dissolved oxygen (DO), which, on a small scale, can be accomplished through the increment of agitation rates with a concomitant increase in the oxygen transfer rate (OTR). On a large scale, e.g., production-scale reactors, it is difficult to the increase in power consumption. The addition of pure oxygen at these scales is expensive and special precautions are required as compressed oxygen is accompanied by safety issues. The ability to demonstrate the possibility to scale up such a process is of great importance and is detrimental to commercial production [35,79].

Kastilan et al. [79] have optimized a fermentation process for the production of two *Pf*AMA1-DiCo-based malaria vaccine candidates, which comprises only two phases: a batch phase and an induction phase. The authors have demonstrated that, even though the induction phase is initiated at a much lower cell density, the process is not only more convenient for the operator and easier to scale up, but it also generates higher product yields with a simultaneously lower level of process-related impurities. Through the use of a new methanol-feeding strategy and increased air pressure instead of pure oxygen supplement, Liu et al. [35] were able to scale up a HCDF, with the production of glycoside hydrolase LXYL-P1-2. This has been accomplished by setting up conditions of the increased air pressure (Ip-Air), developing a new biomass-*stat* methanol-feeding strategy. With this strategy, it was able to reduce the induction DO value to 1% with an initial induction biomass of 100 g L^−1^ (DCW). Their study comprises a solid basis for the commercial preparation of enzymes with the possibility of being applied to the production of other heterologous proteins from the HCDF of *P. pastoris*.

There is a growing interest in the biopharmaceutical industry to enhance production efficiency via shifting batch to continuous manufacturing and this is also valid for processes using *P. pastoris* strains. The expectation is that this will reduce process costs, increasing productivity and product quality. Nonetheless, continuous operation also has its limitations. It requires long-term stability and sterility in the cultures, and a shortening of average long development times. Genetic instability is still a challenge to be addressed when using *P. pastoris* strains in this operation mode. Nevertheless, the cultivation process is product-specific and must be identified on a case-by-case basis with provision for economic constraints [80]. Rahimi et al. [81] has addressed these by optimizing the dilution rates and determining strain-specific parameters, being able to produce a hepatitis B surface antigen (HBsAg) in a continuous fermentation of recombinant *P. pastoris* Mut^+^. They have compared titres, yield, productivity, and specific rates between different dilution rates. Despite the obtained results being in bench-scale fermentation, there is strong insights that the studied recombinant strain could be a suitable candidate for the transition from the fed-batch fermentation process to continuous operation in the production of the recombinant hepatitis B vaccine in the biopharmaceutical industry.

### 3.2. P. pastoris as an Expression System for the Production of Human Subunit Vaccines

**Figure 2:**
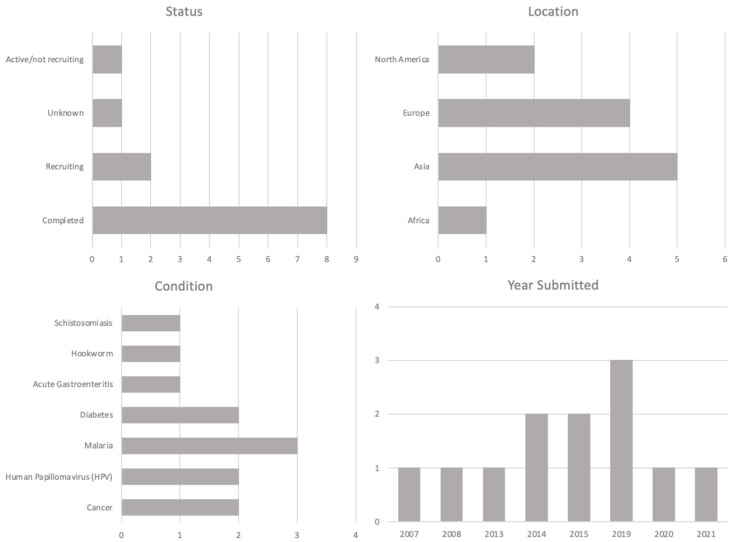
Clinical trials overview using *P. pastoris* as a production platform (clinicaltrials.gov, assessed on 5 August 2021).

Taking into consideration the advantages of this host system along with its genetic manipulation for expression purposes, *P. pastoris* have an important recombinant expression platform of commercial importance. In the last few decades, a broad range of recombinant protein antigens synthesized in *P. pastoris* have been used to develop human sub-unit vaccines against a wide range of diseases, including the ones caused by bacteria and viruses. This type of vaccine is not only considered to be significantly safer than live attenuated and inactivated/killed vaccines, but also likely lends itself to be scaled up in a more cost-effective manner compared with other types of vaccines. In the figure below, an overview of vaccines using *P. pastoris* in clinical trials is shown (Figure 2).

Some other examples of research, i.e., pre-clinical and clinical studies that produced and evaluated vaccines using *P. pastoris*, are discussed in the following sub-sections.

#### 3.2.1. Examples of General Diseases

The most common cause of dementia, accounting for 60–80% of dementia cases, is Alzheimer’s. This disease at a first stage leads to the loss of memory and later progresses to other cognitive abilities (i.e., speech, ability to reason, and movement), which seriously interferes with daily life. There are still no effective treatment options for the great majority of patients, therefore there is a need to continuously fight this hurdle. An example of that is the study performed by Tan and co-workers [82], a recombinant peptide vaccine (r4 × Aβ15) designed to elicit a specific Aβ immune response in the absence of a T cell response with the aim to successfully reduce Alzheimer’s disease (AD), was produced using *P. pastoris.* The authors findings suggested that r4 × Aβ15 was an effective immunogen in C57BL/6 mice, without an autoreactive T cell response, indicating that r4 × Aβ15 may have potential as a safe and effective AD vaccine and can be produced at a low cost.

Epstein-Barr virus (EBV) disease, despite being ubiquitous in the human population worldwide, is a disease against which there is no specific treatment or vaccine. Even though most infections with EBV go unnoticed, in some cases, it can be linked to the development of serious conditions (i.e., cancer). Research is underway to further understand EBV-associated medical conditions and to develop an effective treatment. Wang and co-workers [83] showed the suitability of *P*. *pastoris* for high-level expression of the C-terminal of Epstein-Barr virus recombinant EBNA1 protein (E1ΔGA). The results have shown that the recombinant E1ΔGA elicited strong humoral and cellular immune responses, suggesting that the yeast-expressed E1ΔGA retained good immunogenicity. These achievements were possible through codon optimization, a prerequisite for large-scale industrial production of a subunit vaccine candidate. Another example is the study of the Epstein-Barr virus envelope glycoprotein gp350 as an attractive candidate for a prophylactic vaccine. The results showed that the yeast-expressed gp350 retained strong immunogenicity, providing a useful source for developing an EBV subunit vaccine candidate [84]. A recombinant VP1 protein expressed in *P. pastoris* have shown to induce protective immune responses against human enterovirus 71 (EV71) in mice. The results demonstrated that the yeast-expressed VP1 protein retained good immunogenicity and was a potent EV71 vaccine candidate [85]. EV71 is one of the major agents causing hand, foot, and mouth disease (HFMD). This disease is also associated with serious neurological diseases in children and is highly prevalent in the Asia Pacific regions. Another example of such pivotal findings is the work of Zhang et al. [86, where the authors successfully demonstrated a simple, high-yield production of EV71 VLPs in transgenic *P. pastoris*, lifting a major limitation in commercial development of VLP-based EV71 vaccines.

*Helicobacter pylori* (*H. pylori*) infection is associated with various gastric diseases, and it is known to be a risk factor for gastric cancer (GC). Due to the reduced efficacy of current standard therapies, exploring new strategies for the development of more efficient treatment is vital. The efficacy of alkyl hydroperoxide reductase (AhpC) and mannosylated AhpC (mAhpC) as candidate’s vaccine for *Helicobacter pylori* infection was evaluated by O’Riordan et al. [87]. The authors have cloned, over-expressed, and purified recombinant AhpC and glycoengineered mAhpC using *P. pastoris* as a host system. Their results showed that AhpC confers protection against *H. pylori* infection, which is enhanced when using glycoengineering form mAhpC. The latter represents a promising candidate vaccine against *H. pylori* infection.

Globally, approximately half a million people are chronically infected with hepatitis virus. Viral hepatitis is now one of the leading causes of death worldwide and represents a growing burden for health systems in lower- and middle-income countries. In order to overcome this concern, new treatment approaches in the vaccination field are underway, with the aim of developing an effective and cost-effective vaccine. Gurramkonda et al. [88] presented a detailed and enhanced method for the purification of high-level intracellular production of recombinant surface antigen of the hepatitis B virus (HBsAg) virus-like particles (VLPs) in *P. pastoris*. These results showed that *P. pastoris*-derived HBsAg VLPs exhibited a high potential as a superior biosimilar vaccine against hepatitis B in comparison with the results of mice vaccinated with a gold standard vaccine (Engerix™, GlaxoSmithKline UK Limited, Brentford, UK). Cai and co-workers [47] used *P. pastoris* yeast to express a high-level of truncated hepatitis C virus (HCV) E1E2 protein. Their results have shown that the expressed protein can efficiently induce anti-E1E2 antibodies in rabbits, with the ability to neutralize pseudo-type particles and virions derived from HCV genotype 1a/1b and 2a, respectively. These findings indicate that the recombinant E1E2 glycoprotein is effective in inducing broadly neutralizing antibodies and is a potent HCV vaccine candidate.

Infection by human hookworm, *Necator americanus,* affects hundreds of millions of people living in the poorest regions in the world. Therefore, an effective vaccine for preventing hookworm infection would constitute an important public health breakthrough. Based on these facts, Curti et al. [89], have optimized and revised the production process of the *Necator americanus* glutathione s-transferase 1 (Na-GST-1), the lead hookworm vaccine recombinant protein candidate. Their findings showed that, by adding a sorbitol pulse and co-feed during methanol induction, adjusting the downstream process by tuning the capacity of an ion-exchange chromatography, and altering the hydrophobic interaction chromatography conditions, it is possible to scale up the production for initial phase 1 clinical testing. Furthermore, from the same author is the expression at a 20 L scale and purification of the extracellular domain of the *Schistosoma mansoni* TSP-2 recombinant protein, a vaccine candidate for human intestinal schistosomiasis, an infectious disease that affects more than 230 million people worldwide [90]. In this study, the authors, described the cloning and the expression of the external loop of Sm-TSP-2 recombinant protein secreted by Pichia Pink using a 20 L scale fermentation, and the two-steps purification, which resulted in a protein recovery yield of 31% and a protein purity of 97%. Like in the previous study, the possibility to scale up the production for initial phase 1 clinical testing was demonstrated.

Cervical cancer caused by human papillomavirus (HPV) is the fourth most common cancer in women, with a large majority occurring in less developed regions. Despite the existence of treatments for the health problems that HPV can cause, there is no treatment for the virus itself. Ways of overcoming this limitation have been explored; an example of that is the work of Coimbra et al. [91]. In their study, the authors demonstrated the production of L1 protein from different types of HPV in *P. pastoris* using an integrative vector. The use of such a vector led to improvements on clone stability, making this heterologous expression system a potential tool, subjected to optimization, to produce a VLP-based vaccine. Another example is the study performed by Sanchooli et al. [92], where the authors have developed virus-like particles (VLPs) comprising the HPV-16 L1 capsid protein as a possible tool for prophylactic vaccination. From the results, high expression of HPV-16 L1 gene leading to pure HPV-16 L1 VLP recombinant protein was successfully generated using *P. pastoris* cells, reaching a yield of 11 mg/L. The fact that *P. pastoris* was used as an expression system makes this platform cost-effective for LMICs, contrary to the existent licensed VLPs-based vaccines known as gardasil (Merck and Co., Inc., Kenilworth, NJ, USA) and cervarix (GlaxoSmithKline, London, UK), which, despite being highly efficient, have high-cost production.

Poliomyelitis, a devastating paralytic and sometimes fatal infection, was responsible for global epidemics in the last century. Despite there being no cure for Polio, the disease is preventable with vaccination, namely, by using live-attenuated oral poliovirus vaccine (OPV) and the inactivated poliovirus vaccine (IPV). There are significant safety concerns for the continued use of these vaccines, making it imperative to develop alternative ones. Virus-like particles (VLP) formed by empty capsids produced in *P. pastoris* are presented as a model system for enterovirus VLP vaccine production [93].

#### 3.2.2. Examples of Tropical Diseases

Dengue disease is an emerging mosquito-borne viral infection with increasing reports of outbreaks, and with 2.5 billion people potentially at risk. The global impact of infectious diseases like this is obvious, therefore the vital role of a vaccine in such situations. For the first time, Brata et al. [94] presented the feasibility of secreting dengue virus (DENV) type 2 envelope domain III using *P. pastoris*, demonstrating the relevance of sub-unit approaches to dengue vaccine development. This was possible after exploring various induction parameters for the fed-batch cultivation, including media composition, temperature, pH, and methanol concentration. Moreover, it was shown that this host was also able to produce DENV VLPs with the promise of efficacy and safety, offering key advantages from the perspective of inexpensive vaccine production in developing countries where dengue is endemic [95]. An example of that is the work performed by Rajpoot et al. [96], where the authors described the co-expression and co-purification strategy adopted to obtain tetravalent mosaic VLPs (T-mVLPs) and presented data on the comparison of their immunogenicity with other tetravalent E-based VLP formulations. The obtained results were a proof-of-concept that incorporation of all four E proteins into T-mVLPs does not compromise the immunogenic potential of the individual monomers. Furthermore, it can contribute to further cost reduction as it entails a single expression and purification, making this vaccine prototype an alternative to the next generation dengue vaccine candidates.

Over the last decade, chikungunya virus (CHIKV) has emerged in most parts of the world. Despite its global presence, no licensed vaccine or antivirals are currently available, which creates one of the important public health challenges. For the first time, Saraswat et al. [97] presented the potential of *P*. *pastoris* to express chikungunya virus-like particles (CHIK-VLPs). CHIK-VLPs were found to be immunogenic in mice and efficient in inducing virus neutralizing antibodies and balance Th1/Th2 response. These findings showed the protective efficacy of CHIK-VLPs against the emerging chikungunya virus. Another example of emergent illnesses is Chagas disease, a parasitic disease caused by a protozoan parasite (*Trypanosoma cruzi*). It is estimated that 56,000 new infection cases and 12,000 deaths are registered annually, leading to an urgent need for prophylactic and therapeutic vaccines development. Having these facts in mind, Matos and co-workers [98] developed a prime-boost immunization strategy with Tc52 *N-*terminal domain DNA and the recombinant protein expressed in *P. pastoris*, showing the protective effect against *Trypanosoma cruzi* infection. This strategy, combining DNA and protein vaccine, induced strong cellular and humoral responses as well as specific mucosal IgA, thus conferring better protection in the acute and chronic stages of infection and showing improvements on vaccine efficacy.

Malaria disease is a serious tropical disease that, if not diagnosed and treated promptly, can be fatal. Currently, no vaccine available offers protection against malaria; the treatment relies on antimalarial medication which is strain-dependent. Moreover, resistance to antimalarial medicines has been a recurring problem. Thus, there is an urgent need to find new and affordable approaches to tackle the disease. Taking this into consideration, Jacob and co-workers [99] developed a platform where whole *P. pastoris* yeast-expressing measles virus nucleoproteins were used as a production and delivery system to multimerize *Plasmodium* antigens, which is a major issue in the success of vaccines. In this study, circumsporozoite protein (CS) multimerization is obtained by fusing the CS from *Plasmodium berghei* (*Pb*) ANKA strain to the nucleoprotein (N) protein from measles virus (MV), generating recombinant *P. pastoris* yeast-expressing N or PbCS alone, or *N-*PbCS ribonucleoprotein rods (RNPs), and characterized the size, shape, and cellular localization of these RNP structures. With the aim of providing a means of delivering multi-antigen cocktails in a vaccine formulation, whole recombinant yeast clones, each expressing relevant *Plasmodium* antigens for the asexual and sexual stages, were mixed. With this strategy, the authors demonstrated a low-cost production process, independent from cold chain constraints for vaccine delivery in developing countries. Another interesting approach is the study presented by Spiegel et al. [100]. In this study, the authors optimized a multi-stage, multi-subunit malaria vaccine candidate for the production in *P. pastoris* by the identification and removal of protease cleavage sites. They clearly demonstrated that the proteolytic degradation of recombinant proteins by endogenous *P. pastoris* proteases can be prevented by the identification and removal of such cleavage sites (i.e., KEX2-protease consensus motif EKRE), which is of high importance for product yield and stability and vital for most therapeutic molecules.

#### 3.2.3. Disease Outbreaks and Pandemics

The constant adaptation of microbes and their ability to evolve and become resistant to antibacterial and antiviral agents ensures that infectious diseases will continue to be an ever-present and ever-changing threat. Moreover, changes in human behavior, population growth, easiness to travel, and the emergence of new and resurgent pathogens contributes to the increment of infectious disease rates. Furthermore, it can lead to disease outbreaks that can easily cross borders and threaten economic and regional stability. We have been witnessing the latter historically through HIV, influenza H1N1 and H5N1, SARS, and, more recently, Ebola and COVID-19 epidemics and pandemic, respectively [101,102]. More than ever, there is an urgent need and commitment to work together to accelerate the development and production of new vaccines, and hence to assure equitable access worldwide. Examples of studies to tackle limited treatment options and lack of rapid and cost-effective response strategies for the pathogen outbreaks listed previously are described next.

It is not a surprise that it is hard to generate effective antibody responses to HIV. Nonetheless, recent studies have shown that some vaccines can indeed induce anti-HIV antibody responses. An example is the study presented by Aw et al. [103], whereby the authors demonstrated a systematic analysis of the expression of the anti-HIV VRC01 antibody in *P. pastoris* through signal peptide optimization. Broadly neutralizing antibody (bNAb) VRC01 is currently undergoing Phase II clinical trials to determine its effectiveness in preventing HIV infection. In this study, the authors set out a strategy to increase the production of the bNAb VRC01 by exploring a combinatorial library of signal peptides and evaluating the impact of using a 2A signal peptide to produce both chains of the antibody from a single bicistronic vector.

The spread of influenza A virus results in seasonal pandemics of human respiratory disease, which causes serious public health issues and economic burdens, as mentioned previously. The creation of an efficient vaccine is highly important to be used in an outbreak to support the eradication of the virus, due to the availability to boost immunity and decrease virus shedding into the environment. Having this in mind, Wang and co-workers [104], developed a universal influenza vaccine, by exploiting *P. pastoris* expression system to produce the A/Brisbane/59/2007 HA stem as the antigen. The authors have compared the yield and immunogenicity of the HA stem protein produced in yeast and mammalian (HEK 293T) cells, and the results showed that immunization of mice with the mono-glycosylated form of the H1 stem protein expressed in yeast can elicit greater antibody responses associated with strong neutralization activity. Pietrzak et al. [105] demonstrated that an avian influenza H5N1 virus vaccine candidate based on the extracellular domain produced in *P. pastoris* system as subviral particles protects chickens from lethal challenge. In this current study, it was shown that deletion of multi-basic cleavage site (MBCS) in the extracellular region of the H5 antigen improves protein oligomerization. Further, it was demonstrated that such a modified antigen is more immunogenic than HA with MBCS and protects from a lethal challenge with the H5 subtype of highly pathogenic avian influenza (HPAI).

From 2002 to 2003, severe acute respiratory syndrome (SARS), a respiratory disease caused by SARS coronavirus (SARS-CoV), has spread fast from continent to continent, resulting in 8000 infections, with approximately 10% mortality, and a devastating effect on the economies of the affected areas. In order to ameliorate the effects of future outbreaks, Chen and co-workers [106] have optimized the production process and characterization of the yeast-expressed SARS-CoV recombinant receptor-binding domain (RBD219-N1) as a platform for a SARS vaccine candidate. The authors were able to increase the fermentation (10 L scale production) yield 6- to 7-fold to a 400-mg/L fermentation supernatant (FS). Additionally, their results indicated that the process is reproducible and that the purified, tag-free RBD219-N1 protein has high purity and a well-defined structure, thus making it a suitable candidate for production under current good manufacturing practice and future phase-1 clinical trials.

Ebola virus disease (EVD) is a severe, often fatal illness affecting humans. In the period between 2013–2016, the world has witnessed one of the most widespread outbreaks with a case fatality rate of 40% and a disrupting economy effect. With the aim of overcoming the limited treatment options and to accelerate the production of therapeutics against future pathogen outbreaks, Purcell et al. [107] proposed an efficient development cycle using glycoengineered *P. pastoris* to produce functional anti-Ebola antibodies. In this study, the authors, have engineered a landing pad system into Pichia GlycoSwitch and used the resulting strain to produce the constituent antibodies of the anti-Ebola ZMapp cocktail. This cocktail has shown to be a promising treatment, as it has demonstrated efficacy in nonhuman primates, and is currently in human clinical trials.

Pandemics have always shaped human history, and the COVID-19 pandemic that we are living in nowadays is not an exception. With nearly 200 million confirmed cases of people infected globally and almost 4.3 million deaths, this disease caused by SARS-CoV-2 virus has altered daily life for a sustained period of time. In such a global emergency scenario, the unification of scientific efforts is pivotal to ensure access to a safe and affordable COVID-19 vaccine worldwide. This endeavor to speed up the pandemic response is being achieved through the access to COVID-19 tools (ACT) accelerator. The latter is a tool that brings together governments, scientists, businesses, civil society, philanthropists, and global health organizations (e.g., the Bill & Melinda Gates Foundation, CEPI, Gavi, the WHO). Furthermore, COVAX, co-led by Gavi, CEPI, and WHO, the vaccines’ pillar to access ACT, will facilitate the equitable access and distribution to whichever vaccines prove to be effective globally, prioritizing the people most at risk (i.e., LMICs) [108,109,110,111]. An example of a scientific effort is the work performed by Chen and co-workers [112] who developed a severe acute respiratory syndrome (SARS) subunit recombinant protein vaccine candidate based on a high-yielding, *pichia*-engineered, receptor-binding domain (RBD219-N1) of the SARS beta-coronavirus (SARS-CoV) spike (S) protein, which was formulated with aluminum hydroxide. Their findings have shown that this formulation induced high-level neutralizing antibodies against both pseudotyped virus and a clinical (mouse-adapted) isolate of SARS-CoV, conferring fully protection from lethal SARS-CoV challenge in mice. Therefore, this vaccine formulation is under consideration for further development against SARS-CoV and potentially other emerging and re-emerging beta-CoVs, such as SARS-CoV-2. The same authors have also presented the potential for developing a SARS-CoV-2 receptor-binding domain (RBD) recombinant protein as a heterologous human vaccine against coronavirus infectious disease (COVID)-19 [106]. They have accelerated the evaluation of this vaccine into safety pre-clinical testing.

## 4. Some Economic Considerations

The potential cost advantage of using *Pichia* reported in the literature was demonstrated by Coleman 2020, comparing the costs of manufacturing a hypothetical monoclonal antibody mAb produced in a well0understood generalized CHO cell line with that of the same mAb produced in Pichia. In the absence of data published in the open literature, the approximations in the study could be extended to vaccine manufacture.

**Figure 3:**
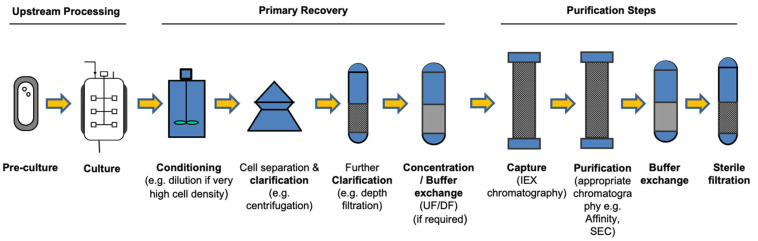
Schematic Overview of a potential process for secreted product.

In terms of process comparability, the main adjustments were the removal of protein A, viral filtration, and deactivation steps. Manufacturing cost of goods (COGs) consisted of raw materials, consumables, labor, and facilities costs. An estimate for upstream costs reductions to 60% of that of CHO cells was given based on shorter culture times and higher cell densities. These differences amounted to ~42% reduction in raw material costs per lot. However, as the authors rightly point out, this only constitutes one part of the overall analysis. The speed of production (including downstream processing) as well as the productivity of the operation and product demand are key factors to consider. Different scenarios were considered. A key finding was the sensitivity of Pichia processing costs to productivity levels, which may need to be improved. There are many ways to improve productivity from cell engineering to continuous processing. These avenues are being pursued and are establishing *Pichia* as a firm contender for therapeutics and vaccines production (Table 2). A typical *Pichia* process is illustrated in Figure 3.

Lowering the overall manufacturing costs is an essential requitement to help LMICs to become more independent. However, it is worth noting that this is only one parameter in defining the needs in such countries. Other political, economic, and social factors are also involved. These are illustrated in Hayman et al., Kraigsley et al., and Phillips et al. [113,114,115].

## 5. Conclusions

The potential of *P. pastoris* in the production of recombinant proteins of commercial interest, as well as subunit vaccines, is immense. The popularity of this host system is due to the various advantages that it can offer, such as stable expression, high-level production, low cost, ease of genetic manipulation, as well as its capability to secrete a heterologous protein in its native conformational structure and strong immunogenicity. Nonetheless, further improvements either related with strain engineering (e.g., engineering of glycosylation) or process engineering (e.g., continuous processing alternative induction systems) will not only strongly increase the productivity of *P. pastoris* system, but also it will expand the range of target proteins including those that are currently only achieved by other expression systems. The rationale behind these strategies for improvement implies considering the endpoint application before deciding the strategy for expression of heterologous proteins and, accordingly, selecting the expression system characteristics. Hence, with the enhancement of the biopharmaceutical application of *P. pastoris* system, we can assume that this host in the coming years will be of prime choice along with, for example CHO cells.

## Figures and Tables

**Table 1:** Comparison of different expression systems for recombinant protein-based biopharmaceuticals production. Adapted from [10,11,12,13].

Host/Platform	Characteristics						Examples of Expressed Products
	Overall cost	Production time	Scale-up capacity	Propagation	Product yield	Contamination risk	Vaccine Candidates
Bacteria	low	low	high	easy	high	medium (e.g., endotoxins)	Hepatitis E virus (HEV) capsid polypeptide
Mammalian cells	high	high	low	hard	medium	very high (e.g., virus, DNA)	Recombinant varicella-zoster virus (rVZV)
Yeast	medium	medium	high	easy	high	low	Hepatitis B surface antigen (HBsAg)
Insect cells	medium	medium	high	feasible	high	low	Truncated dengue envelope proteins (DEN-80E)
Transgenic Plants	medium	medium	high	easy	high	low	Papillomavirus major capsidProtein L1
Transgenic Animals	high	high	low	feasible	high	very high (e.g., virus, DNA)	Malaria major surface protein (MSP-1) antigen

**Table 2:** SWOT analysis of *P. pastoris* platform technology.

Strengths	Weaknesses and Threats	Opportunities
Generally recognized as safe (GRAS ∗ status), robust organism Innate ability to secrete heterologous proteins A highly inducible promoter (alcohol oxidase) that can be easily exploited for recombinant protein production No Crabtree effects Ability to perform certain post-translational modifications Suitable for platform manufacture Very high cell density achievable Low Cost of Goods compared to e.g., mammalian systems Relatively low secreted host cell protein (HCP) Absence of endotoxins/bacterio-phage contamination	Low cellular productivity if not optimised Protease release during fermentation Scale up requires large volume methanol handling and high oxygen input together with substantial heat generation Harvesting/dewatering of very high cell density results in instability in centrifugation	Improved cellular productivity Continuous culture with lower cell concentration and higher productivity, smaller footprint Methanol-free systems

**Table 3:** Examples of expressed antigens against diverse clinical conditions using different yeast species and strategies in which they can be used on vaccine development.

Strategy	Yeast Species	Antigen Immunogen	Disease	Ref
Whole Recombinant Yeast (WRY)	*S. cerevisiae*	HCV NS3-core fusion	Hepatitis C	[17]
	*P. pastoris*	HBsAg, HSP70	Hepatitis B	[18]
Virus Like Particles (VLPs)	*S. cerevisiae*	HBsAg	Hepatitis B	[19]
	*P. pastoris*	DENV envelope protein domain III (EDIII)	Dengue	[20]
	*H. polymorpha*	HPV52L1	Human papillomavirus	[21]
Yeast Display (YD)	*S. cerevisiae*	HIV-1 envelope (Env) glycoprotein	AIDS	[22]
	*P. pastoris*	α-aggulutinin	Avian Influenza virus	[23]
Purified Protein	*S. cerevisiae*	Hemagglutinin-neuramidase	Newcastle disease	[24]
	*P. pastoris*	HCV core E1E2	Hepatitis C	[25]
	*H. polymorpha*	Envelope glycoprotein-E1 ectodomain	Hepatitis C	[26]

Legend: hepatitis C virus (HCV), hepatitis B surface antigen (HBsAg), 70-kDa heat shock proteins (HSP70), dengue virus (DENV), human anti-human papilloma virus 52 late protein L1 (HPV52L1), human immunodeficiency virus type 1 (HIV-1), envelope glycoprotein (Env), hepatitis C virus (HCV).

**Table 4:** Pros and Cons of using *P. pastoris* as an expression system.

Pros	Cons	References
High Expression	Inefficient secretion of larger proteins (>30kDa)	[27,28,29]
Cost-effective	Proteolysis of secreted proteins	[29,30,31]
Relatively rapid growth	Methanol safety requirements (Methanol is highly flammable chemical))	[28,32,33,34]
Scalability	Some glycosylation patterns different from mammalian	[27,28,35,36,37]
Efficient Secretion & Simple purification		[28,38]
Choice of secreted/intracellular expression		[27,28,39]
Efficient protein folding		[27,32]
*N-*glycosylation close to higher eukaryotes (e.g., glycoengineered, GlycoSwitch^®^ (Carlsbad, CA, USA))		[36,37]

**Table 5:** Overall considerations for heterologous protein expression in *P. pastoris* manufacturing optimization [28,40,41].

Strain Engineering					Process Engineering					
Gene Optimisation & Synthesis		Plasmid Construction			Host Strain			Fermentation Mode		
Codon Optimisation	Signal Sequence	Promoter	Selectable Marker	Genomic Integration	Type	Mut Form	Selection	Batch	Fed-Batch	Continuous
Codon usage database	*S.c.* α-MF	Constitutive	Drug resistance	Single multicopy	Wild	+	Microscale cultivation	Carbon source	Induction temperature	Dilution rate (D)
Bioinformatic tools	*P.p.* PHO1	Inducible	Auxotrophy	Homologous recombination	Protease-deficient	-	EasySelect™	Medium composition	Substrate feed rate	
	Kex2p			Ectopic integration	Auxotrophic	s		pH	Specific growth rate (µ)	
	Ste13p				Glyco-engineered			DO	Mixed substrate	

Legend: *Saccharomyces cerevisiae* α-mating factor secretion leader sequence (*S.c.* α-MF), *P. pastoris* acid phosphatase (*P.p.* PHO1), kexin proprotein convertase (KEX2p), dipeptidyl aminopeptidase A (Ste13p).

**Table 6:** Methodologies to enhance downstream processing [12,42,43].

Process Development Methodologies	Application	Examples
High throughput (HT) approaches	Cell lysis	24-well-HT sonication, high pressure homogenisation
	Refolding	Circular dichroism, refolding kits
	Purification	Aqueous two-phase systems, pre-packed Predictor filter plates
	Solubility	Multiscreen assay system, Chromafil^®^ (Loughborough, UK) Multi96 filter plates
Single-use systems	Harvesting	Ksep^®^ (centrifugation) (Surrey, UK), ARTeSYN’s^®^ (TFF) (Kilbarry, Ireland)
Design of Experiments (DoE)	Screen critical process parameters (CPPs) based on critical quality attributes (CQAs)	Jmp, Design-Expert, Modde software’s
Process Analytical Technology (PAT)	Analysis of protein concentration, purity, host cell protein (HCP), host cell DNA (HCD), etc.	Spectroscopy, HPLC, circular dichroism
Continuous DSP processing	Purification	chromatography
	Formulation	Freeze-drying
Integrative system	Upstream and Downstream coupling	InSCyT

## Data Availability

Not applicable.
